# Heritability and Longitudinal Stability of Planning and Behavioral Disinhibition Based on the Porteus Maze Test

**DOI:** 10.1007/s10519-016-9827-x

**Published:** 2016-11-25

**Authors:** Catherine Tuvblad, Marcella May, Nicholas Jackson, Adrian Raine, Laura A. Baker

**Affiliations:** 1 Department of Psychology, University of Southern California (USC), (SGM 501), 3620 S. McClintock Ave, Los Angeles, CA 90089-1061 USA; 2 School of Law, Psychology and Social Work, Örebro University, Örebro, Sweden; 3 Departments of Criminology, Psychiatry, and Psychology, University of Pennsylvania, Philadelphia, USA

**Keywords:** Porteus Maze Test, Planning, Behavioral disinhibition, Heritability, Stability, Executive functions

## Abstract

The Porteus Maze Test (PMT) provides measures of planning and behavioral disinhibition. The PMT was administered to 941 twins during Wave 1 (9–10 years) and 320 twins during Wave 2 (11–13 years). Participants were drawn from the University of Southern California Risk Factors for Antisocial Behavior Study (RFAB). Heritability of behavioral disinhibition, determined by PMT Q-Score, were 33% at Wave 1 and 52% at Wave 2. For planning, determined by Test Age, heritability was 53% at Wave 1; at Wave 2, the non-shared environment was important in boys, whereas genetic influences were important in girls. Both indices were modestly stable (*r* = 0.52; *r* = 0.37). A common genetic factor influenced both indices, respectively, at the two time points, with no ‘new’ genetic variance at Wave 2; the non-shared environment was time-specific. Thus, both genetic and non-shared environmental influences are important for behavioral disinhibition (Q-Score) and planning (Test Age).

**Electronic supplementary material:**

The online version of this article (doi:10.1007/s10519-016-9827-x) contains supplementary material, which is available to authorized users.

## Introduction

The Porteus Maze Test (PMT) was developed in the 1960′s in order to evaluate motor intelligence as a supplement to the Stanford-Binet intelligence test. It was devised by Stanley Porteus as an assessment of planning capacity in a restricted situation, based on the idea that planning is a key element of intelligent behavior (Porteus 1965). Porteus conducted several studies in which the PMT served to differentiate between individuals with higher and lower intellects, resulting in his claim that the PMT is a valid measure of such constructs as planning ability, judgment, foresight, impulsivity, and the ability to delay gratification (Porteus 1965). Additionally, Porteus held that PMT performance was a predictor of social maladjustment, including delinquency and other antisocial behaviors (Porteus 1965).

There are three versions of the PMT: the Vineland revision, the Extension, and the Supplement.^1^ The Vineland revision of the PMT consists of 12 unique maze designs of increasing difficulty. Participants are instructed to complete the mazes by using a pencil to draw a line from the starting point to the endpoint of the maze without lifting the pencil, crossing or bumping into lines, or entering dead ends or blocked alleys. Performance on the measure is gauged by scores on two primary indices: Test Age (TA) and Qualitative Score (Q-Score).^2^ TA is calculated by considering the highest level of maze completed and the number of trials taken to complete each level. Q-Score refers to the quality of test performance as determined by errors in style and strategy, including crossing lines, cutting corners, going in the wrong direction, drawing a wavy line, and lifting the pencil, such that a higher Q-Score reflects a lower quality of performance. Participants are not aware of the qualitative scoring of the measure.

The PMT has been utilized frequently in empirical research; however, there is substantial variation in PMT administration and scoring procedures across studies. Despite numerous revisions, the procedures outlined by Porteus are often confusing and easily misinterpreted, and therefore insufficient for the replication of findings. Articles often cite the Vineland revision of the PMT (as described in Porteus 1965) without offering further elaboration of methodology (e.g., Arán-Filippetti and de Minzi 2012; Deckel et al. 1996; Draper and Ponsford 2008; Fooks and Thomas 1957; Lilienfeld et al. 1996; Purcell 1956). To our knowledge, no published article within the last three decades has provided a clear and consistent set of procedures for PMT administration and scoring. In the present study, we therefore expanded on the Vineland revision of the PMT.

At present, the PMT is generally considered to provide measures of executive functions (Carlozzi 2011) and is often utilized as a predictor of mental anticipation in studies considering executive dysfunction or frontal lobe damage (Krikorian and Bartok 1998; Mack and Patterson 1995). Specifically, the PMT assesses planning (Carlozzi 2011) and behavioral disinhibition (Gow and Ward 1982). TA provides a measure of prehearsal (Porteus 1965), or planning, while Q-Score provides a measure of behavioral disinhibition, or directly impulsive behaviors as they impede planned task execution, including the failure to follow instructions and carelessness (Gow and Ward 1982). Other investigators have identified executive functions as a key element of antisocial and delinquent behavior, and the Q-Score as a robust predictive measure of delinquency (Morgan and Lilienfeld 2000).

The heritability of the specific executive functions of planning and behavioral inhibition has been previously considered, though never as assessed with the PMT. Planning, for example, was assessed with the Stocking of Cambridge task in a sample of Russian twins (mean age: 12.9 years), and the variance in planning was explained primarily by shared (30%) and non-shared environmental factors (63%), with additive genetic factors being of much more limited importance (7%) (Voronin et al. 2016). In a sample of middle-aged male twins, planning was assessed with the number of attempts required in the Tower of London task, and heritability was estimated at 28%, with the remaining variance explained by both shared (17%) and non-shared (55%) environmental effects; this pattern persisted for variables of speed, planning time, and efficiency (Kremen et al. 2009).

Inhibition, assessed with number of errors of commission during a No-Go task in a sample of 7–9-year-old twins, was estimated to be 45% heritable, with the remaining variance attributable to the non-shared environment (Kuntsi et al. 2006). In a sample of 8-year-old twins, the heritability of inhibition as assessed with a modified stop signal task was assessed at 27%, with the remaining variance due to the non-shared environment (Schachar et al. 2011).

Twin studies of other executive functions have generally found heritability to range between 49 and 74%, with the remaining variance explained by non-shared environmental factors (e.g., Polderman et al. 2006; Stins et al. 2004). See Doyle et al. (2005) for an overview of related studies. There is additionally a literature on the heritability of impulsivity, a related construct. A meta-analysis included 41 twin studies of impulsivity with a total of 27,147 participants spanning from infancy to adulthood. Results indicated that equal proportions of the variance within impulsivity were attributable to non-shared environmental (50%) and genetic (50%) influences, with the genetic influences comprised of both additive (38%) and non-additive (12%) effects. Age proved to be a significant moderator, and total genetic effects, although important across all age groups, were most dominant in children (Bezdjian et al. 2011)

In the aforementioned twin studies of planning and inhibition, sex differences were not explored; some studies were limited by their sample (Kremen et al. 2009; Schachar et al. 2011), and others elected to utilize sex-standardized scores (Kuntsi et al. 2006). In the aforementioned studies of other executive functions, however, models accounting for sex differences were examined (Polderman et al. 2006; Stins et al. 2004), and in the meta-analysis on impulsivity, genetic effects were stronger in males than in females (Bezdjian et al. 2011). Relatedly, in a study of decision-making as assessed with the Iowa Gambling Task, no sex differences were found (Tuvblad et al. 2013).

The PMT—among other measures—is a valid measure of the executive functions of planning and behavioral (dis)inhibition across socioeconomic status (Krikorian and Bartok 1998) and culture (David 1974). Test administration is brief, ranging from 10 to 15 min, and requires few materials. The test is inexpensive and can also be administered gesturally, without the use of language (Porteus 1965). Despite these strengths, and despite extensive historical use, the PMT subsists as an underutilized neuropsychological measure (Krikorian and Bartok 1998).

The aims of the present study are twofold: First, data from a large sample of twins participating in the Risk Factors for Antisocial Behavior (RFAB) Twin study (Baker et al. 2013) were used to estimate to what extent genetic and environmental factors influence the executive functions of planning and behavioral inhibition as assessed by the PMT using TA and Q-Score indices at ages 9–10 and 11–13 years. Second, we investigated to what extent genetic and environmental factors influence PMT performance, as well as the stability of PMT performance, between ages 9–10 and 11–13 years.

## Method

### Participants

The data for the present study were drawn from the University of Southern California (USC) Twin Study of Risk Factors for Antisocial Behavior (RFAB). RFAB is a longitudinal study of the interplay of genetic, environmental, social, and biological factors on the development of antisocial and aggressive behavior from childhood to young adulthood. To date, five waves of data have been collected: During Wave 1 (which took place between 2001 and 2004), the twins were 9–10 years old (*mean age* = 9.60, *SD* = 0.59); during Wave 2 (2003–2006) the twins were 11–13 years old (*mean age* = 11.79, *SD* = 0.92); during Wave 3 (2006–2010), the twins were 14–16 years old (*mean age* = 14.87, *SD* = 0.87); during Wave 4 (2008–2011), the twins were 17–18 years old (*mean age* = 17.28, *SD* = 0.77); and during Wave 5 (2011–2015), the twins were 19–20 years old (*mean age* = 19.91, *SD* = 1.37). The twins and their families were recruited through Los Angeles schools and demonstrate diverse demographic characteristics representative of the greater Los Angeles area. Complete details on study protocol, including zygosity determination, can be found elsewhere (Baker et al. 2013).

The PMT was administered during Waves 1 and 2. The present study considers data from 941 participants with complete data on PMT performance from Wave 1. These include 218 monozygotic (MZ) male twins, 218 MZ female twins, 132 dizygotic (DZ) male twins, 148 DZ female twins, and 110 males and 115 females of opposite-sex twin pairs. A subset of these participants (*n* = 320) were administered the Extension series of the PMT during Wave 2 (see below).

### Procedures and measures

The initial Wave 1 participation involved a 6–8 h laboratory assessment consisting of behavioral interviews, neurocognitive testing, and psychophysiological assessment. The PMT Vineland Revision was administered during the neurocognitive testing portion of the first wave assessment and took approximately 15 min to complete. Six months after Wave 1 was completed, 30 families were invited for a retest session. For PMT, a total of 22 twin pairs were retested and the correlation between the two time points was .33 *p* < .05 for TA, and .47 *p* < .05 for Q-Score. Wave 2 follow-up involved a reduced 3–4 h assessment; the PMT Extension series was again administered during the neurocognitive testing portion of this second assessment.

Administration of the PMT at Wave 1 began with maze years V and VI for demonstration and practice of the task, followed by years VII, VIII, IX, X, XI, XII, XIII, XIV, and Adult I. Porteus (1965) deems that practice at the first time of assessment suffices for subsequent assessment, so administration of the Extension series at Wave 2 began without practice at Year VII, which was then followed by years VIII, IX, X, XI, XII, XIII, XIV, and Adult I. Successive levels of the mazes were presented on individual pieces of paper attached to a clipboard and participants were given pencils to complete them.

Due to several ambiguities in the maze test instructions detailed by Porteus (1965), we expanded on the Vineland revision of the PMT, as described in the RFAB Porteus Maze Test Administration Manual in Appendix A. The manual includes an extended version of Porteus’ scoring sheet, which simplifies the calculation of TA and Q-Score. These procedures were followed for the present study. Most importantly, for unambiguous interpretation of the Q-Score (reflecting the number of qualitative errors), *the entire set of mazes must be administered to each subject*. Although Porteus generally recommends stopping administration after discontinue criteria (3 failed years at any point, or 2 successive failures in Year IX +) have been met, he allows for relaxing this rule “when a complete qualitative record is desired” (p. 250). Administration of the same set of mazes to all subjects is required in order for the Q-Score to be comparable across subjects. Otherwise, low Q-Scores could result either from the fact that fewer mazes were administered (for less planful subjects) or from lower error rates (for more planful subjects).

In his original instructions, Porteus assigned a weight ranging from 1 to 3 to each qualitative error (1965). He also added a qualitative error to the Year VI or Year VII maze, depending on PMT version, which amounts to a doubling of qualitative errors made on these mazes. However, Porteus offers no explanations of how the qualitative error weights were derived or why errors made in Year VI or VII deserve additional weight. In our sample, the weighted Q-Score, calculated with both the qualitative error weights and Year VII error, correlated with the non-weighted Q-Score *r* = 0.97 (*p* < 0.05). In the interest of parsimony, further analyses proceeded with the non-weighted Q-Score.

### Statistical analyses

In the classic twin design, covariances between MZ and DZ twins are utilized to separate the variance of a measured trait into genetic and environmental components (Neale and Cardon 1992). MZ twins share a common environment as well as 100% of their genes, while DZ twins share a common environment and only about 50% of their genes. By comparing twin similarity for the measured trait between groups of MZ and DZ twin pairs, the total phenotypic variance of the trait can be divided into additive genetic factors (A), shared environmental factors (C), and non-shared environmental factors (E). Shared environmental factors are non-genetic influences that contribute to similarity within pairs of twins, while non-shared environmental factors are experiences that make siblings dissimilar, including measurement error. Additive genetic factors can be used to estimate heritability as the proportion of total phenotypic variance due to genetic variation. Evidence of the effects that are present is given by comparing the intraclass correlation for MZ and DZ twins (Neale and Cardon 1992). For example, a DZ intraclass correlation approximately half the value of the MZ intraclass correlation would indicate the presence of genetic effects within a given wave, whereas a DZ intraclass correlation more than half a MZ intraclass correlation indicates the presence of both genetic and shared environmental effects. However, this is a descriptive approach and formal modeling is required to achieve accurate estimates.

PMT scores were positively skewed; data were therefore log transformed with the statistical software SAS 9.1.3 (SAS 2002–2004), yielding a more normal distribution.

Univariate genetic models were fit with the structural equation program Mx (Neale et al. 2003) to estimate the relative contributions of A, C, and E to PMT performance using log-transformed scores. The genetic and environmental influences across the two waves of PMT performance were determined for the *n* = 320 participants with PMT data for both Waves 1 and 2 in a bivariate Cholesky decomposition. This method of factorization breaks down the variance and covariance of each PMT score at the two waves (i.e., TA_W1_ and TA _W2_; Q-Score_W1_ and Q-Score _W2_) into A, C, and E factors. The decomposition has the same number of factors in each of the A, C, and E components as the number of observed variables. That is, the first genetic factor loads on the PMT score at both waves, whereas the second genetic factor only loads on the PMT score at Wave 2; this same procedure repeats for the C and E components.

A bivariate Cholesky decomposition was also used to estimate genetic (*rg*) and environmental correlations (*rc, re*). A genetic correlation (*rg*) indicates the extent to which genetic effects on one measure overlap with genetic effects on another measure, in our case, Q-Score and TA within Wave 1 and Wave 2. While a shared environmental correlation (*rc*) and non-shared environmental correlation (*re*) indicate overlap among shared and non-shared environmental factors for the different symptoms. These statistics vary from −1.0 and +1.0, and are independent of the magnitudes of genetic and environmental influence for each set of measures (Posthuma et al. 2003).

Chi squared (χ^2^) tests were utilized to compare goodness of fit between each model and a baseline saturated model, which perfectly captures observed variances, covariances, and means for each twin and zygosity group. The parsimony of the models—based on the balance between model fit and number of parameters—was evaluated with the Akaike Information Criterion (AIC) (Akaike 1987) and the Bayesian Information Criterion (BIC) (Raftery 1995), where lower values indicating better fit.

## Results

### Descriptive statistics

Means and standard deviations of the untransformed PMT scores as well as twin correlations for the log-transformed scores are shown in Table 1. In Wave 1, there was no significant difference in mean or variance of Q-Score across zygosity (male MZ vs. DZ *t*_(463)_ = 1.64, *p* = 0.10; female MZ vs. DZ *t*_(474)_ = 1.76, *p* = 0.08) or sex (*t*_(946)_ = 1.53, *p* = 0.13), and there was no significant difference in mean or variance of TA across zygosity (male MZ vs. DZ *t*_(469)_ = 1.87, *p* = 0.06; female MZ vs. DZ *t*_(479)_ = 0.88, *p* = 0.38) or sex (*t*_(957)_ = 1.77, *p* = 0.08).

**Table 1:** Means (SD), number of participants (n) and twin correlations^a^ for PMT performance, by sex and zygosity

	Males		Females		DZ opposite sex	
	MZ	DZ	MZ	DZ	Males	Females
Means (standard deviations)						
Wave 1 Q-Score	41.90 (17.46) n = 218	44.72 (20.61) n = 132	39.86 (17.41) n = 218	42.17 (18.23) n = 148	44.89 (20.21) n = 115	43.85 (21.66) n = 110
Wave 1 TA	13.04 (2.50) n = 223	12.60 (2.58) n = 133	12.64 (2.73) n = 221	12.29 (2.62) n = 150	12.58 (2.73) n = 115	12.60 (2.76) n = 110
Wave 2 Q-Score	31.51 (16.00) n = 77	33.30 (16.51) n = 44	30.95 (15.26) n = 88	30.54 (13.88) n = 48	30.13 (15.69) n = 30	32.63 (15.52) n = 30
Wave 2 TA	14.39 (2.22) n = 78	14.00 (2.17) n = 44	13.91 (2.99) n = 80	13.73 (2.40) n = 50	14.68 (2.32) n = 30	13.55 (2.30) n = 30
Twin correlations						
Wave 1 Q-Score	0.44*	0.40*	0.57*	0.26*	0.45*	
Wave 1 TA	0.48*	0.35*	0.55*	0.17*	0.30*	
Wave 2 Q-Score	0.55*	0.08	0.57*	0.25	0.20	
Wave 2 TA	0.48*	0.59*	0.40*	0.06	0.58*	

^a^Means and SDs are for raw data, while twin correlations are for log-transformed Q-Score and TA

* *p* < 0.05

In Wave 2, there was no significant difference in mean or variance of Q-Score across zygosity (male MZ vs. DZ *t*_(149)_ = − 0.19, *p* = 0.85; female MZ vs. DZ *t*_(156)_ = − 0.17, *p* = 0.87) or sex (*t*_(308)_ = 0.42, *p* = 0.67), and there was no significant difference in mean or variance of TA across zygosity (male MZ vs. DZ *t*_(150)_ = 0.75, *p* = 0.06; female MZ vs. DZ *t*_(158)_ = 0.59, *p* = 0.56) or sex (*t*_(311)_ = 1.90, *p* = 0.058).

Twin correlations for the log-transformed PMT scores in Table 1 give a first indication of genetic and environmental influences for executive functions as measured by TA and Q-Score. With regard to Q-Score at Waves 1 and 2, MZ correlations were slightly higher than DZ correlations, suggesting the influence of both genetic and shared environmental influences. Similarly for TA at Wave 1, MZ correlations were slightly higher than DZ correlation, suggesting the influence of both genetic and shared environmental influences. For Wave 2, the pattern was less clear, perhaps due to the smaller sample size at the follow-up assessment.

### Univariate genetic model fitting

Univariate genetic model fitting results within each measurement time-point are summarized in Table 2. For both Q-Score and TA within Wave 1, a full ACE model fit the data well in comparison to the saturated model. This model could be reduced without significant loss of fit by constraining parameters between males and females to be equal (Q-Score Wave 1: Δ*χ* ^2^ = 0.86, *df* = 3, *p* = 0.83; TA Wave 1: Δ*χ* ^2^ = 1.53, *df* = 3, *p* = 0.68).

**Table 2:** Univariate genetic results for PMT performance, ages 9–10 and 11–13 years

	Overall fit							*χ* ^2^ difference test			Parameter estimates (95% CI)		
Variable	−2LL	Df	AIC	BIC	*χ* ^2^	df	*p*	Δ*χ* ^2^	df	*p*	A	C	E
Wave 1 Q-Score													
Saturated model	−557.33	924	−2405.33	−3167.93									
ACE males ≠ females	−547.82	933	−2413.82	−3191.32	9.51	9	0.39						
ACE males = females	−546.96	936	−2418.96	−3200.27	10.37	12	0.58	.86	3	0.83	0.33 (0.05–0.60)	0.22 (0.00–0.43)	0.46 (0.37–0.56)
AE males = females	−541.55	937	−2415.55	−3200.69	15.78	13	0.26	5.41	1	0.02			
CE males = females	−543.60	937	−2417.60	−3201.72	13.73	13	0.39	3.34	1	0.07			
E males = females	−446.01	938	−2322.01	−3156.05	111.32	14	<0.001	100.95	2	<0.001			
Wave 1 TA													
Saturated model	4460.50	935	2590.50	−695.21									
ACE males ≠ females	4467.44	944	2579.44	−719.90	6.94	9	0.64						
ACE males = females	4468.97	947	2574.97	−728.52	8.47	12	0.75	1.53	3	0.68			
AE males = females	4468.97	948	2572.97	−731.65	8.47	13	0.81	0	1	0.99	0.53 (0.44–0.61)	–	0.47 (0.39–0.56)
CE males = females	4482.59	948	2586.59	−724.84	22.09	13	0.05	13.62	1	<0.001			
E males = females	4551.26	949	2653.26	−693.63	90.76	14	<0.001	82.29	2	<0.001			
Wave 2 Q-Score													
Saturated model	−134.820	292	−718.82	−818.94									
ACE males ≠ females	−131.709	301	−733.71	−840.55	3.11	9	0.96						
ADE males ≠ females	−132.666	300	−732.67	−838.46	2.37	8	0.97						
ACE males = females	−131.449	304	−739.45	−848.14	3.37	12	0.99	.26	3	0.97			
AE males = females	−131.449	305	−741.45	−850.72	3.37	13	0.99	0	1	0.99	0.52 (0.35–0.65)	–	0.48 (0.35–0.66)
CE males = females	−124.833	305	−734.83	−847.41	9.99	13	0.69	6.62	1	0.010			
E males = females	−103.891	306	−715.89	−839.51	30.93	14	0.01	27.56	2	<0.001			
Wave 2 TA													
Saturated model	3006.70	612	1782.70	−414.42									
ACE males ≠ females	3022.41	621	1780.41	−434.76	15.71	9	0.07				0.00^a^	0.15	0.85
											(0.00–0.42)	(0.00–0.43)	(0.56–1.00)
											0.55^b^	0.00	0.45
											(0.18–0.72)	(0.00–0.28)	(0.28–0.72)
ADE males ≠ females	3031.12	620	1791.12	−427.27	24.42	8	0.002						
ACE males = females	3028.21	624	1780.21	−441.26	21.51	12	0.04	5.80	3	0.12			
AE males = females	3028.21	625	1778.21	−444.40	21.51	13	0.06	0	1	0.99			
CE males = females	3032.44	625	1782.44	−442.28	25.74	13	0.02	4.23	1	0.04			
E males = females	3039.39	626	1787.39	−441.94	32.69	14	0.003	11.18	2	0.004			

*-2LL* -2(log-likelihood); *AIC* Akaike’s Information Criterion; *BIC* Bayesian Information Criterion

*χ* ^2^ difference in log-likelihoods between nested models; *df* change in degrees of freedom; *A* additive genetic variance

*C* shared environmental variance; *E* non-shared environmental variance; *a* boys; *b* girls

For Q-Score at Wave 1, based on AIC and BIC it was not clear whether the full ACE male equal to female model could be further reduced, as such the results of the full ACE male equal to female model are presented here. Genetic influences explained 33% (*p* < 0.05) of the variance, shared environment 22%, and the non-shared environment accounted for the remaining 46% (*p* < 0.05) of the variance. For TA at Wave 1, the full ACE model with estimates equated across males and females was further reduced by dropping the shared environment (W1: Δ*χ* ^2^ = 0.00, *df* = 1, *p* = 1.00) (W2: Δ*χ* ^2^ = 0.00, *df* = 1, *p* = 1.00). Genetic influences accounted for 53% (*p* < 0.05) of the variance, with non-shared environmental effects accounting for the remaining 47% (*p* < 0.05) of the variance.

For Q-Score and TA within Wave 2, low DZ twin correlations were detected, in boys for Q-Score and in girls for TA, Table 1. This may be due to non-additive genetic effects, such as epistasis or dominance (Neale et al. 2003). A model estimating additive genetic (A) effects, non-additive genetic (D) effects and non-shared environmental (E) effects was therefore first tested. Based on AIC and BIC, the full ACE model (Model 2) was found to fit better than the ADE model.

For Q-Score within Wave 2, a full ACE model fit the data well in comparison to the saturated model. This model could be reduced without significant loss of fit by constraining parameters between males and females to be equal (Q-Score Wave 2: Δ*χ* ^2^ = 0.26, *df* = 3, *p* = 0.97). The full ACE males equal to females model could be further reduced by dropping the shared environment (Δ*χ* ^2^ = 5.41, *df* = 1, *p* = 0.02). Genetic influences accounted for 52% (*p* < 0.05) of the variance, with non-shared environmental effects accounting for the remaining 48% (*p* < 0.05) of variance. For TA at Wave 2, as the DZ male correlation was significant and higher than the MZ male correlation (Table 1), we present the results from the full ACE model. For boys, the non-shared environment primarily accounted for the variance 85% (*p* < 0.05). For girls, genetic influences accounted for 55% (*p* < 0.05) of the variance, with non-shared environmental effects accounting for the remaining 45% (*p* < 0.05) of variance.

### Bivariate genetic model fitting

For both Q-Score and TA within Wave 1, the phenotypic correlation was *r* = − 0.64 (*p* < 0.0001), and within Wave 2 the phenotypic correlation was *r* = − 0.62 (*p* < 0.0001). The results of the bivariate genetic modeling within Waves are presented in Table 3. Within Wave 1, a full ACE model provided a better fit to the data than the saturated model (AIC = −6636.14, BIC = −7301.12). The model was further reduced by equating parameters across males and females (Δ*χ* ^2^ = 8.86, *df* = 9, *p* = 0.45) and by dropping the shared environment (Δ*χ* ^2^ = 4.33; *df* = 3; *p* = 0.23). The genetic correlation between TA and Q-score in Wave 1 was *rg* = 0.81 (95% CI 0.71–0.90) and the non-shared environmental correlation was *re* = 0.41 (95% CI 0.30–0.51). It should be noted that the non-shared environmental correlation (re) could also reflect correlated measurement error between the two measures. Within Wave 2, a full ACE model provided a good fit to the data relative to the saturated model (*χ* ^2^ = 52.84; *df* = 48; *p* = 0.29). The model was further reduced by equating parameters across males and females (Δ*χ* ^2^ = 21.98, *df* = 9, *p* = 0.01) and by dropping the shared environment (Δ*χ* ^2^ = 5.19; *df* = 3; *p* = 0.16). However, based on AIC the ACE males ≠ females model fit the data better, whereas based on BIC and AE males = females model better described the data. This may partly be explained by the small sample size at Wave 2. As such, the genetic and environmental correlations should be interpreted with caution. The genetic correlation between TA and Q-score in Wave 2 was *rg* = 0.68 (95% CI 0.48–0.82) and the non-shared environmental correlation was *re* = 0.59 (95% CI 0.45–0.71).

**Table 3:** Bivariate Genetic Results for PMT Performance, Ages 9–10 to 11–13 Years

	Overall fit							*χ* ^2^ difference test		
	-2LL	Df	AIC	BIC	*χ* ^2^	df	*P*	Δ*χ* ^2^	df	*P*
Q-Score—TA Wave 1										
Saturated model	−2960.36	1823	−6606.36	−7184.04						
ACE males ≠ females	−2894.14	1871	−6636.14	−7301.12	66.22	48	0.04			
ACE males = females	−2885.28	1880	−6645.28	−7324.85	75.08	57	0.06	8.86	9	0.45
AE males = females	−2880.95	1883	−6646.95	−7332.07	79.41	60	0.05	4.33	3	0.23
Q-Score—TA Wave 2										
Saturated model	−1052.20	551	−2154.20	−1944.24						
ACE males ≠ females	−999.36	599	−2197.36	−2041.36	52.84	48	0.29			
ACE males = females	−977.38	608	−2193.38	−2053.53	74.82	57	0.06	21.98	9	0.01
AE males = females	−972.19	611	−2194.19	−2058.65	80.02	60	0.04	5.19	3	0.16
AE males ≠ females	−989.96	602	−2193.96	−2044.38	62.24	51	0.14	9.40	3	0.02
Q-Score Waves 1 to 2										
Saturated model	−709.84	1194	−3097.84	−4135.89						
ACE males ≠ females	−737.39	1228	−3193.39	−4257.33	27.55	34	0.77			
ACE males = females	−733.05	1237	−3207.05	−4283.66	23.22	43	0.99	4.34	9	0.89
AE males = females	−730.51	1240	−3210.51	−4291.89	20.67	46	0.99	2.55	3	0.47
AE males = females, drop A22, E21	−727.16	1242	−3211.16	−4296.54	17.32	48	0.99	5.90	2	0.05
TA Waves 1 to 2										
Saturated model	−2682.43	1208	−5098.43	−5168.66						
ACE males ≠ females	−2668.65	1242	−5152.65	−5269.50	13.780	34	0.99			
ACE males = females	−2648.00	1251	−5150.00	−5287.69	34.43	43	0.82	20.65	9	0.01
AE males = females	−2645.88	1254	−5153.88	−5296.13	36.60	46	0.84	2.13	3	0.55
AE males = females, drop A22, E21	−2643.25	1256	−5155.25	−5301.15	39.18	48	0.81	4.75	2	0.09

-*2LL* -2(log-likelihood); *AIC* Akaike’s Information Criterion; *BIC* Bayesian Information Criterion

*χ* ^2^ difference in log-likelihoods between nested models, *df* change in degrees of freedom

Further, Q-Score and TA showed significant longitudinal stability between Waves: *r* = 0.52 (*p* < 0.0001) for Q-Score and *r* = 0.37 (*p* < 0.0001) for TA. The results of the bivariate longitudinal genetic modeling are also presented in Table 3. For both Q-Score and TA, a full ACE model provided a better fit to the data than the saturated model (Q-Score: *χ* ^2^ = 27.55; *df* = 34; *p* = 0.78) (TA: *χ* ^2^ = 13.78; *df* = 34; *p* = 0.999). In both cases, the model was further reduced by equating parameters across males and females (Q-Score:Δ*χ* ^2^ = 4.34, *df* = 9, *p* = 0.89) (TA: Δ*χ* ^2^ = 20.65, *df* = 0, *p* = 0.01) and by dropping the shared environment (Q-Score: Δ*χ* ^2^ = 2.55; *df* = 3; *p* = 0.47) (TA: Δ*χ* ^2^ = 2.13; *df* = 3; *p* = 0.55). These models could be further reduced by dropping non-significant estimates A22, E21 (Q-Score: *χ* ^2^ = 5.90; *df* = 2; *p* = 0.52) (TA: *χ* ^2^ = 4.75; *df* = 2; *p* = 0.93).

**Fig. 1:**
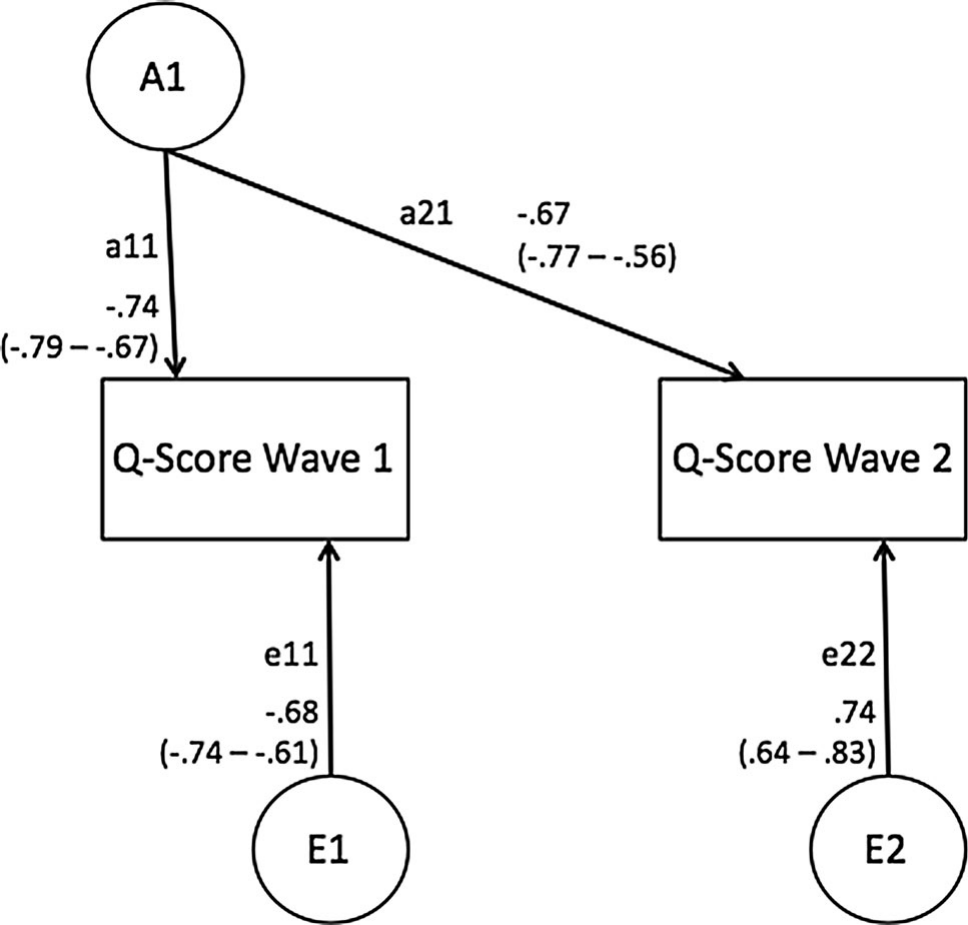
Bivariate Longitudinal Genetic Model of Q-Score, Ages 9–10 to 11–13 Years

**Fig. 2:**
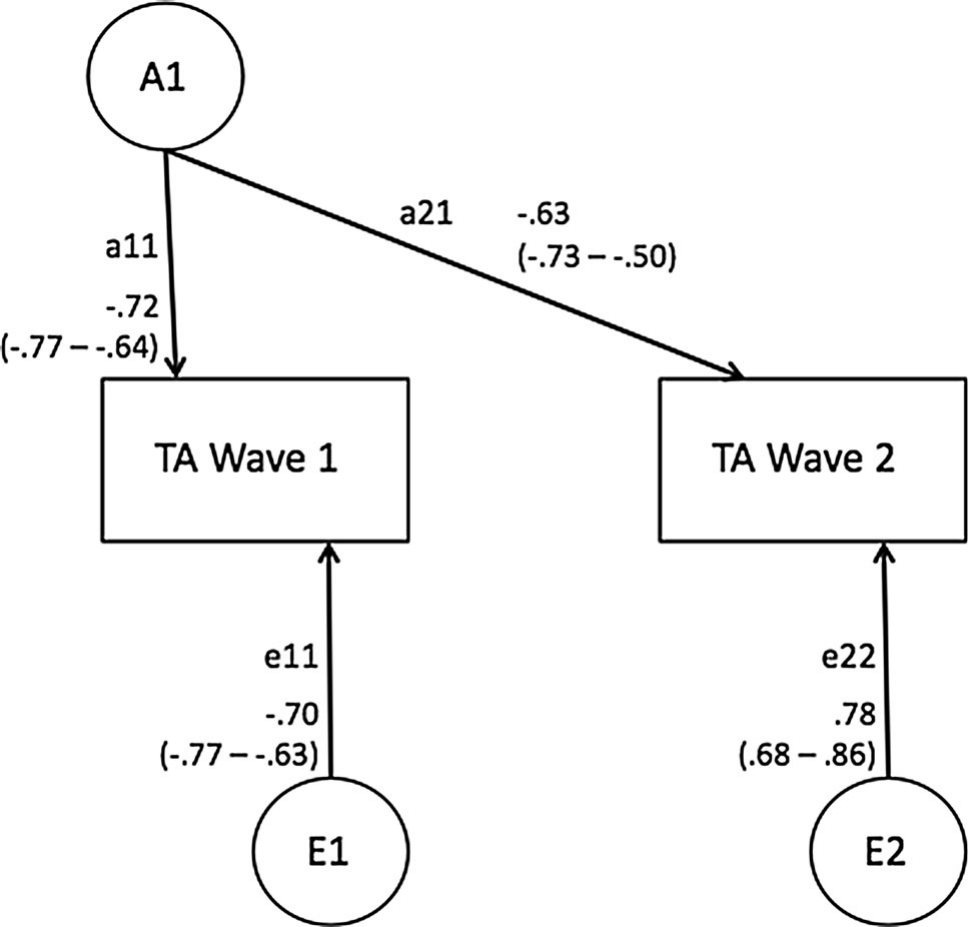
Bivariate Longitudinal Genetic Model of TA, Ages 9–10 to 11–13 Years

Figures 1 and 2 display estimates from the best-fitting bivariate longitudinal models. The total estimated genetic and environmental effects for TA and Q-Score at each time point can be obtained by summing the contributions of common and unique components. The estimated heritability in TA at Wave 1 was (a_11_)^2^, i.e., 0.72^2^ = 0.51, and at Wave 2 (a_21_)^2^ + (a_22_)^2^, i.e., .63^2^ + 0.0^2^ = 0.40. Importantly, a single genetic factor influenced TA at both Waves, with no significant ‘new’ genetic variance appearing for TA at Wave 2. The influence of the non-shared environment on TA was specific to each time of assessment: 0.49 at Wave 1 and 0.61 at Wave 2. The estimated heritability in Q-Score at Wave 1 was (a_11_)^2^, i.e., 0.74^2^ = 0.54, and at Wave 2 (a_21_)^2^ + (a_22_)^2^, i.e., .68^2^ + 0.0^2^ = 0.45, again with a single genetic factor influencing both assessments. The influence of the non-shared environment was again time-specific, 0.46 at Wave 1 and 0.46 at Wave 2. In general, the total genetic and environmental effects estimated for each measure in a bivariate Cholesky decomposition are consistent with those derived in a univariate genetic model. Slight variation in the parameter estimates are a result of additional information available in cross-twin cross-age covariance.

## Discussion

The PMT assesses the executive functions of planning and behavioral (dis)inhibition across socioeconomic status (Krikorian and Bartok 1998) and culture (David 1974). Administration is brief, inexpensive, and does not require the use of language. The strengths of the PMT warrant its continued application. The goal of this study was to investigate to what extent genetic and environmental factors influence Q-Score and TA at ages 9–10 and 11–13 years. Approximately one-third of the variance in Q-Score, a measure of behavioral disinhibition, was the result of genetic contributions, with the remaining variance attributable to non-shared environmental factors. Study results further indicated that during childhood (age 9–10 years) approximately one half of the variance in TA, a measure of planning, was the result of genetic contributions, while the remaining variance was found to be attributable to non-shared environmental factors. Administering the PMT Extension series in a follow-up assessment of a subset of the original cohort of twins approximately two years later (age 11–13 years) yielded similar results for Q-Score, with one half of the variance in Q-Score, or behavioral disinhibition and the remaining variance was attributable to non-shared environmental factors. For TA, non-shared environmental factors were important for boys, whereas genetic factors were important for girls. Further, not all genetic influences were common for Q-Score and TA within each of the two waves. This was indicated by the genetic correlations between these measures being less than one. A non-overlapping genetic variance suggests that Q-Score and TA are somewhat independent in their underlying biological substrates.

The present study sought to investigate to what extent genetic and environmental factors influence PMT performance longitudinally between ages 9–10 and 11–13 years. Phenotypically, based on mean values, average Q-Score seemed to decrease, whereas average TA seemed to increase between the two measurement occasions. This is as expected, since children’s command of executive functions is presumed to increase across childhood and adolescence, i.e., both planning and behavioral inhibition are expected to develop over time, with decreasing Q-Scores and increasing TA across age.

The longitudinal results revealed that both Q-Score and TA, or behavioral disinhibition and planning, were modestly stable between the two time points, suggesting that the rank-order of individual remains relatively similar across age. The stability across Wave 1 and Wave 2 was explained by a common genetic factor for each index, whereas the influence of the non-shared environment was found to be time-specific for both Q-Score and TA. This indicates that variance in behavioral disinhibition and planning within each measurement occasion was partly due to non-shared environmental factors, which include idiosyncratic experiences for each twin as well as measurement error.

It is unclear whether sex differences predominate in the heritability of planning and behavioral disinhibition. Despite that most models fit to the data presented in this paper equated parameters between males and females, the small sample size and resulting low power make it difficult to draw conclusions. In fact, in the case of the bivariate model, AIC and BIC contradicted one another in this respect. Previous heritability studies of executive functions diverge regarding whether or not they find evidence for sex differences (Polderman et al. 2006; Stins et al. 2004; Bezdjian et al. 2011); thus, further research to clarify this point, at least as regards planning and behavioral disinhibition, would certainly add to the literature.

### Limitations

The results of this study are subject to several potential limitations. First, the sample size at Wave 2 was relatively small, which resulted in larger standard errors and confidence intervals for genetic and environmental effects. It is also possible that we would have been able to detect significant shared environmental influences with a larger sample. The replication of our findings in larger samples with more statistical power is therefore important. Second, there are several assumptions in the classical twin design that may not have been met. If the equal environment assumption is not met, higher correlations among MZ twins may be due to environmental factors rather than genetic factors and heritability may be overestimated. However, studies examining the equal environment assumption generally report that it holds and that the resulting bias is likely modest (Felson 2014). Additionally, weights as described by Porteus (1965) were not applied to qualitative errors in the present analyses, nor were Year VI or Year VII weighted errors included. As previously stated, weighted and unweighted Q-Scores were highly correlated in our sample, and we recommend the parsimonious use of the latter, particularly since Porteus did not offer any rationale for applying the weights. Also, practice and age effects could have influenced the testing results between Wave 1 and Wave 2, see mean values Table 2. Thus, it is possible that PMT performance is influenced by prefrontal cortex maturation and cognitive development (Tuvblad et al. 2013).

## Conclusion

This study examined the heritability and longitudinal stability of PMT Q-Score and TA, indices of planning and behavioral disinhibition, in a sample of twins at age 9–10 and age 11–13. Analyses revealed genetic and non-shared environmental influences on both of these executive functions. Furthermore, results indicated that the stability of these functions between the ages of 9–10 and 11–13 is primarily due to genetic influences, with no ‘new’ genetic variance emerging across this narrow window of development.

## Electronic Supplementary Material

Below is the link to the electronic supplementary material.

Supplementary material 1 (PDF 1768 kb)

## Footnotes

1 The Extension and Supplement were devised in order to eliminate practice effects in situations where the experimenter would like to repeat the test; they are supplemental, rather than alternative, to the original series.

2 The PMT also provides a third index, the Test Quotient (TQ), which is a ratio of the subject’s chronological age and the subject’s TA, with scores below 30 or above 135 considered “of little comparative significance” (Porteus 1965, p. 255). 35.2% of our subjects obtained a TQ of 135 and 50.8% of subjects obtained a TQ above 130. We therefore believe that the TQ is of little significance in this sample, and have looked to TA and Q-Score in consideration of PMT performance.
