# Supplementary material for: Heritability and Longitudinal Stability of Planning and Behavioral Disinhibition Based on the Porteus Maze Test
Source: Behav Genet. 2016 Nov 25;47(2):164–74. doi: 10.1007/s10519-016-9827-x (PMC5306271; doi:10.1007/s10519-016-9827-x)
Supplement: Supplementary file 1 — Supplementary material 1 (PDF 1768 kb) [file 10519_2016_9827_MOESM1_ESM.pdf]

The RFAB
Porteus Maze Test Administration Manual

Risk Factors for Antisocial Behaviors
University of Southern California

The Porteus Maze Test

Stanley Porteus developed the Porteus Maze Test (PMT) in the early twentieth century as a supplement to the Stanford-Binet Intelligence Test. It was devised in order to assess planning capacity in a restricted situation, based on the idea that prehearsal is a key element of intelligent behavior (Porteus, 1965).

The administration procedures outlined by Porteus (1965), however, are often vague and easily misinterpreted. To our knowledge, no article published within the last three decades has provided a consistent manner of interpreting these procedures, making it problematic to replicate research methodology. Utilizing the original administration procedures (1965) as a template, the authors of this manual aim to clarify and expand on ambiguous points of maze administration and scoring procedure.

Porteus, S. D. (1965). *The Porteus Maze Test: Fifty years' application*. Palo Alto, CA: Pacific Books.

***Changes and additions to these instructions are presented in italics.*

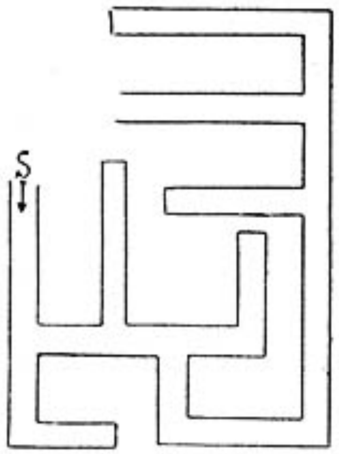

Strengths

- PMT performance appears to be a valid indicator of planning and behavioral disinhibition across socioeconomic status (Krikorian & Bartok, 1998) and culture (David, 1974).
- The PMT can be administered gesturally, without the use of language
- Administration of the PMT is inexpensive and requires few materials
- Administration time is between 10 and 15 minutes

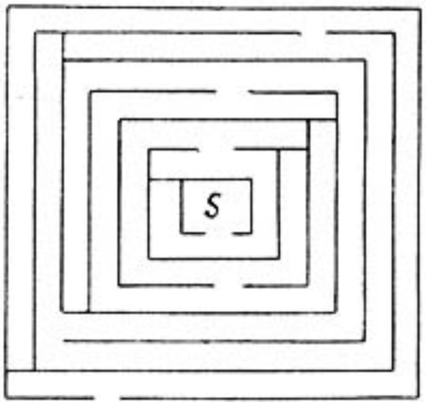

Manual Contents

Overview	2
Beginning, End, and Inversion Trials	3
Administration Instructions	4
Qualitative Score	5
Test Age	8
Test Quotient	10
Scoring Sheets	11

Correspondence to Marcella May
mfhmay@gmail.com

3260 S McClintock Ave, Dept. of Psychology SGM 501
Los Angeles, CA 90089

2016

Overview

The PMT, broadly considered, yields scores on indices that are utilized as measures of executive functions.

The PMT consists of a series of eight or twelve mazes, depending on the version administered.

Mazes are labeled as years, each of which corresponds to one or two years of age. They are administered successively, increasing in difficulty.

PMT Versions

1.	Vineland Revision (Original Series)	Year III – Year XII, Year XIV, Adult I
2.	Extension	Year VII – Year XII, Year XIV, Adult I
3.	Supplement	Year VII – Year XII, Year XIV, Adult I

The additional versions of the PMT are supplemental to, rather than alternative to, the original series. The second and third versions were devised to eliminate practice effects in situations where the experimenter would like to repeat the test.

Performance Indices

The subject is instructed to find his or her way out of the given maze. Two aspects of the subject's performance are indexed: the subject's success in his or her progression through the trials of the maze years (via Test Age and Test Quotient) and the subject's errors in style and strategy throughout performance (via Qualitative Score). The errors affecting a subject's score on these indices are termed Test Age Errors and Qualitative Errors, respectively.

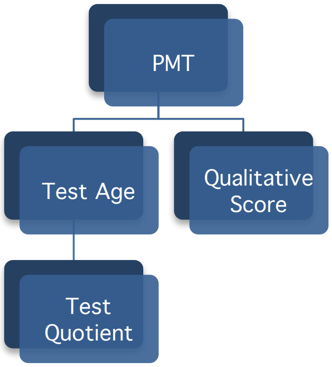

1. Test Age (TA) is calculated with the weight of the highest maze year the subject successfully completed and the number of trials required to complete each year. It is used as a measure of planning or foresight (Carlozzi, 2011).

2. Qualitative Score (Q-Score) is calculated qualitatively by considering the number of errors in style and strategy. It is often considered as a measure of behavioral disinhibition (Gow & Ward, 1982).

3. Test Quotient (TQ) is a ratio of a subject's chronological age with that subject's TA. As such, it is used to measure a subject's planning ability in relation to that subject's age.

Maze Trials

Year III – Year IV	3 Trials*
Year V – Year XI	2 Trials
Year XII+	4 Trials

Unsuccessful trials are those in which Test Age Errors are committed. If the number of trials allotted for a given maze year have been administered and all scored as unsuccessful, that maze year is recorded as a failure.

*These trials are generally administered for practice purposes, as discussed on the subsequent page. According to Porteus, credit may be allotted for these trials if fewer than three line crossings occur on any one trial.

Beginning, End, and Inversion Trials

If a maze year is failed but the next highest year is passed, an inversion is administered for the higher year. Discontinue criteria (for the purposes of TA scoring) are met after 3 failed years in the series or 2 successive failed years in Year IX+

Where to Begin

In the Vineland Revision…

- Subjects younger than age 6 and those with developmental disabilities begin with Year III. All others begin with Year V.
- Year V and Year VI can be used for practice "for timid or withdrawn subjects, with primitive individuals or [in] special cases where the language used in instructions is a barrier to understanding" (p. 246)

** *We propose that Year V and Year VI can be used for practice with all subjects.*

Rationale: "In the Original series for more sophisticated older children not under any suspicion of mental defect, [Year VII] is the starting point of the maze test application" (p. 249). Thus, scoring does not begin until Year VII.

- Subjects should go on to Year VII only when Year VI is performed correctly and without help.

In the Extension and Supplement…

- Administration always begins with Year VII and no practice or demonstration is allowed, since the subject will already have completed the original series.

Where to End

In all Versions…

- Discontinue criteria are met after 3 failed years at any point in the series *or* 2 successive failed years in Year IX+
- Porteus states that administration of further mazes will cease once the discontinue criteria have been met, but also that the series of mazes can be administered to completion for additional qualitative scoring

  ***We recommend that the full set of mazes be administered to all subjects, and that the discontinue criteria only be used in the calculation of the TA score.*

  Rationale: A subject that meets discontinue criteria during administration may have a lower qualitative score than a subject who completes all the mazes, since the discontinued subject would have been presented fewer maze trials.

Inversions

- Occur when:
  1. The subject has failed all trials of a maze of a particular test year
  2. The subject passes the next highest test year within the allotted trials
- Inversion: an additional trial of the same maze year that the subject has just passed, rotated 180°
  - Administered to avoid scoring accidental success
  - The same number of trials allotted for that same (non-inverted) maze year is allowed for the subject to complete the inverted maze
- If it occurs:
  - <u>If the subject is able to successfully complete the inverted maze in the allotted number of trials</u>, that maze year is passed
  - <u>If the subject is unable to successfully complete the inverted maze in the allotted number of trials</u>, that maze year is scored as a failure, even though the subject was able to pass that year in the regular (non-inverted) trials.

Administration Instructions

Rapport should be established before testing begins.

Examiner and subject should sit at a table, with the examiner seated across from the subject if possible.

The surface of the table should be smooth and a pencil of medium bluntness should be provided.

The examiner should hold the top of the test design with his or her fingers so that it cannot move

***A clipboard may be used for the same purpose*.

The subject may not trace over the maze design with his or her pencil or finger.

The examiner may not indicate the opening of the maze at the end of Year VIII or above. If the subject asks, the examiner should say, "You must find your own way out."

Verbal Instructions

Year III: "I want to see whether you can draw all around between these lines without crossing or touching them with the pencil. You draw just like this." (Examiner demonstrates a correct, slow, and careful drawing performance between guidelines from the arrow to just around the first angle, then removes this maze and replaces it with a new, blank version of the same design. The examiner encourages the child wherever possible, and can guide the child hand-over-hand if necessary.)

Year IV: "Do this the same way. Begin here (Examiner indicates starting arrow) and draw right around without crossing any of the printed lines." (After the second trial, the examiner may indicate to the subject where he or she touched or crossed lines)

Year V: "This is what is called a maze and you must draw with your pencil like this (Examiner draws approximately 1.5 inches starting from the arrow near the rat and around the first turn). These lines are all supposed to be walls and the rat went in here (Examiner points to arrow) to try and get some cheese (Examiner points to cheese at end of maze). Now, I want you to draw a line showing me where the rat went to find the cheese. But, you must be careful not to cross any lines, *bump into any walls or go into any place that is a dead end* or is blocked at the other end. If you go into any blocked space, you cannot turn around and come out. You must start all over again with a new maze. *This is not a speed test.* One more thing you must remember- you can stop anywhere as long as you like *while you decide which way to go*, but try not to lift your pencil until you have drawn right to the end of the maze."

Year VI: "This is another maze. Begin here and show me where the rat went to get the cheese. But do not cross any lines or go into any blocked places"

Year VII: Do NOT read instructions for subjects who have already worked through simpler mazes. Otherwise: "I want you to suppose that this is a maze in the form of a street map. All the lines are stone walls. You can imagine, if you like, that you are walking or driving a car in here (Examiner points to starting point marked S) and you have to find our way out here (Examiner points to exit arrow). But you must be very carful not to bump into any of the walls nor go into any blocked street, because if you do so you cannot turn around or back out. So if you go into a blind street, you must start all over again. This is not a speed test. You can stop anywhere as long as you like while you decide which way to go, but try not to lift the pencil off the paper until you are right outside the maze, and don't bump into any walls. Start as soon as you are ready.

Years VIII, IX, and X: "Begin here and find your way out." (Examiner points to starting arrow only)

Years XI, XII, and XIV, and Adult I: "Begin here in the center and find your way out."

Years XII and XIV, and Adult I: "Begin here in the center and find your way out."

Test Age

TA is affected by the number of trials it takes the subject to successfully complete each year of the mazes.

Any error resulting in an unsuccessful trial is termed a Test Age Error.

If a subject commits a test age error, the examiner removes the maze from the subject.

There are three types of Test Age Errors.

Test Age Errors

1. Blind Alley

- Occurs when:
  1. The subject enters a blind alley or blind street…
     - Blind alley or blind street: an area of the maze that is blocked at the end, in which the subject would have to turn around and retrace his or her steps to continue in the proper direction
  2. …"By the width of an imaginary penciled line blocking off the entrance" (p. 247)
     ***The width of the imaginary penciled line is defined as equal to the width of the printed walls of the maze*
- Judging whether the error has been committed:
  1. Has a blind alley or street been entered?
     - If the subject self-corrects corrects before entering the blind alley by more than the width of a pencil line, this is scored as the qualitative error of 'wrong direction' (described below) rather than a test age error
     - Pencil slips do not always result in a blind alley test age error
       → If the pencil slips and enters a blind alley *through the opening to that blind alley*, the result is a test age error (see Figure 1a)
       → If the pencil slips and does not enter a blind alley through the opening to that blind alley, only a qualitative error of 'crossed line' (described below) would be scored, provided that the subject returns to the point where the pencil slipped to continue drawing (see Figure 1b).

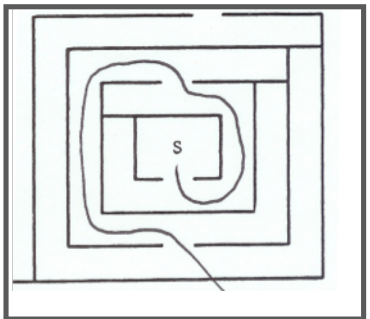

Fig. 1a: The subject's pencil slipped and entered a blind alley through the opening to that blind alley.

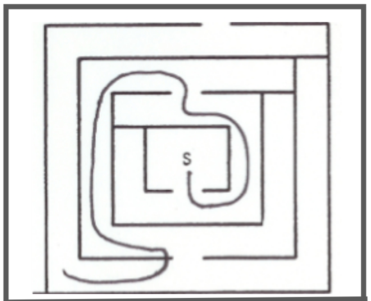

Fig. 1b: The subject's pencil slipped but did not enter a blind alley through the opening to that blind alley. This is not a test age error.

  2. …By the width of an imaginary penciled line?
     - Examiner holds a straight edge to the edge of the printed maze line farthest into the blind alley
       → If *any* of the subject's pencil line shows beyond the straight edge, the trial is unsuccessful (see Figure 2a)
     - If a blind alley does not have a printed maze line, the examiner holds a straight edge to the tip of the subject's drawn line and visually compares the distance by which the alley has been entered (marked "A" on Figure 2b) to the thickness of the printed walls
       → If the distance of "A" is greater than the thickness of the printed walls, the maze is scored as unsuccessful

2. Cut Alley

- Occurs when:
    1. The subject cuts across from one alley to another to reach an open space, in order to avoid drawing all the way around
- Judging whether the error has been committed:
    1. Has the subject cut across an alley?
        - If the subject crosses the printed line before the tip, leaving any of the printed line protruding beyond the subject's pencil line, this is scored as a cut alley
        - A cut alley can occur at any point within the maze, including at the beginning of a maze as the subject exits the box in the center of the maze, and as the subject exits the maze.
        - A cut alley cannot occur while turning around a 90-degree corner. This would not be scored as a test age error, but rather as the qualitative error of 'cut corner' (described below).

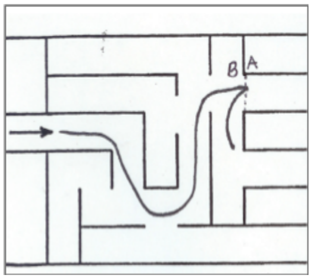

Fig. 2a: The dotted line represents the straight edge; notice that it is aligned with the side of the printed line farthest into the blind alley, marked "A". This maze is unsuccessful because the pencil line goes beyond the straight edge.

3. Inability to Complete the Maze

- Occurs when:
    1. The subject says, "There is no way out"
    2. The subject pauses for 5+ seconds *or* lifts his or her pencil
- Judging whether the error has been committed:
    1. Has the subject said, "There is no way out"?
        - This instruction is taken literally, such that the subject must say nearly verbatim "There is no way out" Examiner holds a straight edge to the edge of the printed maze line farthest into the blind alley
            - → Statement made by the subject must indicate that he or she is unable to find the exit of the maze
        - Statements indicating frustration or noncompliance rather than an inability to find the exit are qualitatively different from an inability to complete the maze and therefore not deserving of a test age error
            - → E.g., "I can't do this" or "I don't want to do this anymore"
            - → In this case, the examiner can prompt the subject to continue if it is deemed necessary
    2. Has the subject paused for 5+ seconds *or* lifted his or her pencil?

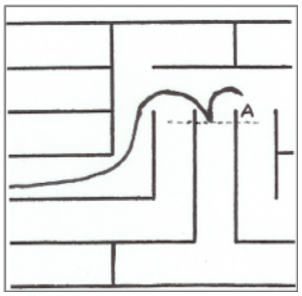

Fig. 2b: The examiner should compare the length of "A", which is the length by which the subject has entered the blind alley, to the thickness of the printed maze walls. In this case, "A" is clearly longer than the thickness of the printed maze walls, thus this maze is unsuccessful.

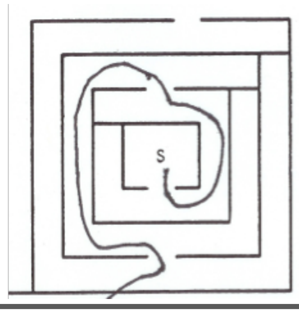

Fig. 3: The subject's line avoids drawing through the maze opening by drawing through a printed line. This is a test age error and the trial is scored as unsuccessful.

Calculating TA

The maximum TA score a subject can obtain is the TA of the most difficult maze the subject was administered:

Up through Year XI, each maze year corresponds to its equivalent TA

Year VII = 7, Year VIII = 8, Year IX = 9, Year X = 10.

As of Year XII, each maze year corresponds to two TA years

Year XII = 12 and 13, Year XIV = 14 and 15, Adult 1 = 16 and 17

If a subject was administered all maze years:

TA = 17 - (½ (# unsuccessful trials pre-Discontinue)* + ½ (max # trials post-Discontinue)*)

* For each maze year, use the worse score between ordinary and inversion administrations

TA score is affected by unsuccessful trials, meeting discontinue criteria, and administration of inversion trials.

- Unsuccessful trials:
  - ½ year is deducted from the maximum TA score for every unsuccessful trial
- Meeting discontinue criteria:
  - The maximum deduction is given for every trial after discontinue criteria are met
    - Maximum deduction = ½ (# of trials allowed)
  - Trials preceding the fulfillment of discontinue criteria are scored normally, with ½ year deducted per unsuccessful trial
- Years in which inversions are administered:
  - "The worse performance of the 2 presentations, ordinary [regular] or inverted, is recorded for scoring purposes" (p. 247)
  - Thus, if both regular trials and inversions are administered for a particular year, the set of mazes in which the subject required more trials to successfully complete the year is used for TA scoring

    ***Porteus does not specify if he is referring to TA scoring or qualitative scoring, so we have taken this to refer to TA scoring only*

PORTEUS MAZE SCORE SHEET

Maze Year	# Reg Trials Administered:	# Inverted Trials:	# Pencil Lifts (Reg)	# Pencil Lifts (Inv)	Failed Year?	Deductions
V	1					
VI	1					
VII	(1) 2		/		(N) Y	(0) ½ 1
VIII	(1) 2	1 2	0		(N) Y	(0) ½ 1
IX	(1) 2	1 2	ll		(N) Y	(0) ½ 1
X	1 (2)	1 2	llll		(N) Y	0 (½) 1
XI	1 (2)	1 2	ll		N (Y)	0 ½ (1)
XII	1 2 3 (4)	1 2 3 4	卌 l		N (Y)	0 ½ 1 1½ (2)
XIV	(1) 2 3 4	(1) 2 3 4	l	lll	(N) Y	0 ½ 1 1½ (2)
Adult	(1) 2 3 4	1 2 3 4	lll		(N) Y	0 ½ 1 1½ (2)

Max. Score: 17 Deductions: 7 ½

Test Age (Max. Score – Deductions): 9 ½

<u>TA Scoring Example:</u>

This subject passed mazes from Year VII – Year IX on the first trial, Year X on the second trial, failed Year XI and Year XII, and passed Year XIV and Adult I on the first trial; the subject's TA score is 9 ½.

Breakdown: Discontinue criteria were met in Year XII, therefore ½ year is deducted from the maximum TA score of 17 for each unsuccessful trial in Year XII and below. At ½ point per unsuccessful trial, 1 unsuccessful trial in Year X + 2 unsuccessful trials in year XI + 4 unsuccessful trials in Year XII contribute to a total deduction of 3½. Every year after the discontinue criteria are met receives the maximum deduction. Thus, the deduction for Year XIV and Adult I is 4.

The total number of deductions for the test administration, 3 ½ + 4, is 7 ½. This is subtracted from the maximum score of 17, resulting in a TA score of 9 ½.

Qualitative Score

Qualitative scoring of the PMT is intended to "reveal any haphazard, impulsive, or overconfident habits of action, or a tendency to... neglect other directions for executive performance" (p. 253).

There are seven types of Qualitative Errors.

Qualitative Errors

1. First Third (FT)

- Occurs when:
  - A subject fails a maze, by entering a blind alley or by cutting an alley, in the first third of that maze
    - Porteus uses an arrow to indicate the blind alley located at the end of the first third of each maze (p. 271-296)
    - A subject would receive an FT error if he or she failed the maze by entering the blind alley denoted by the first arrow, or if he or she failed the maze at any point before drawing past the first arrow
  - Only one FT can occur per maze

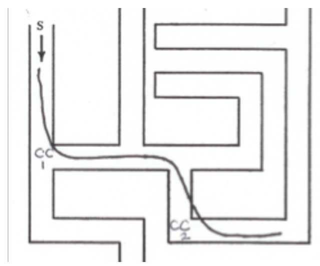

Fig. 4a: Cut corner touching the corner (CC1) and cut corner cutting off the corner (CC2).

2. Last Third (LT)

- Occurs when:
  - A subject fails a maze, by entering a blind alley or by cutting an alley, in the last third of that maze
    - Porteus also uses an arrow to indicate the blind alley located at the beginning of the last third of each maze (p. 271-296)
    - A subject would receive an LT error if he or she failed the maze by entering the blind alley denoted by the last arrow, or if he or she failed the maze at any point after drawing past the last arrow
  - Only one LT can occur per maze

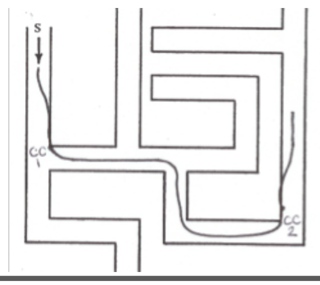

Fig. 4b: Cut corner beginning before corner has been turned (CC1) and cut corner continuing after corner has been turned (CC2).

3. Cut Corner (CC)

- Occurs when:
  - The subject's pencil line touches a printed maze line while turning a 90° corner (see Figure 4a, CC1) *or* cuts off a 90° corner while turning around it (see Figure 4a, CC2)
    - A CC error can begin far before the corner is reached (see Figure 4b, CC1) or it can continue after the corner has been turned (see Figure 4b, CC2)
    - As long as the pencil line does not pull away from the printed maze line, this is only a CC error

4. Crossed Line (CL)

- Occurs when:
  - The subject's pencil line touches a printed line in the maze other than while turning a 90° corner (see Figure 5)

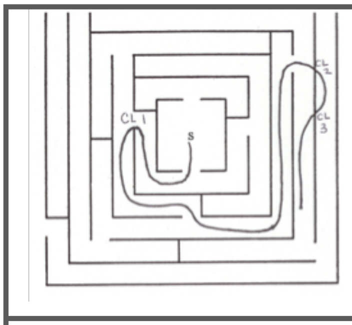

Fig. 5: Examples of CL

- Scored strictly, such that if even the smallest amount of the pencil line touches the printed line, a crossed line should be scored.
  - → If the pencil line touches the printed maze line, pulls away, and then touches the line again, a crossed line should be scored at each point that the pencil line touches the printed line, as long as a defined white space can be seen between the two points of contact

5. Lift Pencil (LP)
- Occurs when:
  - The subject lifts his or her pencil off of the paper, except after exiting the maze
  - Each time the pencil is lifted, an LP occurs

6. Wrong Direction (WD)
- Occurs when:
  - The subject starts to enter a blind alley, but changes direction to continue in the proper direction before entering the alley by more than the width of a pencil (See Figure 6a)
    - WD errors and blind alley Test Age Errors are mutually exclusive, yet similar, events
      - → If the subject crossed into the blind alley by *more* than the width of a pencil, this would be scored as a blind alley test age error and the trial considered unsuccessful (See Figure 6b)
    - If the subject drew close to a blind alley, but never considered entering it, no WD would be scored
      - → This adds an element of subjectivity; the examiner must make a judgment about the subject's intent based on observation of the subject's progress through the maze

    ***We recommend that the examiner make a mental note of WD's as the subject progresses through the maze and then write "WD" on the maze at the locations of the WD's immediately after taking the maze from the subject*

7. Wavy Line (WL)
- Occurs when:
  - The subject's pencil line is irregular or wavy at any point on a maze (see Figure 7)
    - Scored strictly, such that any small bump, dip, or shaky line would result in a WL being scored for that maze
    - The subject's line should be straight and smooth to avoid scoring a WL
      - → It is not necessary for corners to be turned with a 90° angle; corners turned with a smooth curve are acceptable
  - Only one WL can be scored per maze

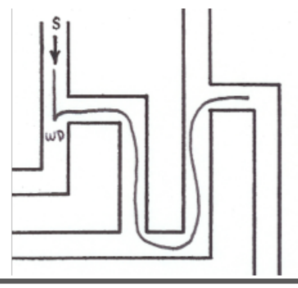

Fig. 6a: The subject self-corrects his or her path before entering the blind alley; this is scored as WD.

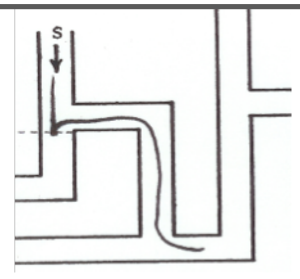

Fig. 6b: The subject does not self-correct before entering the blind alley and therefore receives a test age error rather than WD.

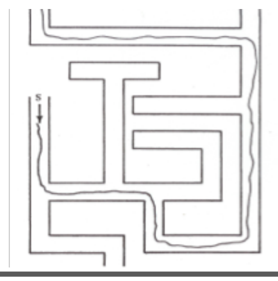

Fig. 7: Wavy performance throughout the maze

Calculating Q-Score

Q-Score is calculated once the subject has completed the entire series of mazes

- The Q-Score is the total number of error points over all mazes administered, including both regular trials and inversions
  ***Each qualitative error receives one error point.*
  Rationale: see below
- The abbreviation for each error should be written at the point on the maze where the error occurred, with the following exceptions:
  - The WL error, which is scored considering the entire maze performance and can thus be written anywhere on the maze
  - LP errors, which should be silently counted while the subject is drawing and totaled at the top of each maze design once the subject has finished

Weighted Q-Score and the Year VI/VII Error

Q-Score is calculated once the subject has completed the entire series of mazes

- Porteus assigned a weight for each qualitative error, ranging from 1 to 3 (p. 253 to 254)
- Porteus additionally adds a qualitative error that is scored only on the Year VI or Year VII mazes:
  - Year VI/VII Error = total number of Qualitative Errors made on all Year VI/VII mazes
    → This effectively doubles the number of Qualitative Errors for Year VI or Year VII

  ***We recommend that, in the interest of parsimony, weights as described in Porteus (1965) not be applied to the Q-Score*
  Rationale: Porteus offers no explanation of how the qualitative error weights were derived or why errors made in Year VI or Year VII deserve additional weight. In our sample the weighted Q-Score, calculated with both the qualitative error weights and Year VII error, correlated with the non-weighted Q-Score r=0.97 ($p < .05$).

Test Quotient

Test Quotient (TQ) is a ratio of chronological age and TA.

Tables to assist in TQ calculation are provided in the appendices of Porteus (1965).

These tables allow cases to obtain a maximum TQ of 135 at each age level.

***In our experience, TQ provides little information in addition to TA and Q-Score in indexing executive functions.*
Rationale: Porteus states that TQ's "below 30 and above 135 are of little comparative significance" (p. 255). In our sample, 35.2% of subjects obtained a TQ of 135 and 50.8% of subjects scored 131 or higher. This offers little variability in the scores; therefore, the upper end of the quotient becomes truncated. While consideration of TQ was not beneficial to our study, this may be due to the narrow age range of our sample size. Virtually no current research uses TQ in analyses.

Scoring Sheets

Q-Score

	FT	LT	CC	CL	LP	WL	WD	TOTAL
Year VII -Regular								R
Year VII -Inversion								I
Year VIII -Regular								R
Year VIII -Inversion								I
Year IX -Regular								R
Year IX -Inversion								I
Year X -Regular								R
Year X -Inversion								I
Year XI -Regular								R
Year XI -Inversion								I
Year XII -Regular								R
Year XII -Inversion								I
Year XIV -Regular								R
Year XIV -Inversion								I
Adult I -Regular								R
Adult I -Inversion								I
TOTAL								

TA and TQ

Maze Year	# Trials Administered:	# Inverted Trials:	# Pencil Lifts (Reg)	# Pencil Lifts (Inv.)	Failed Year?	Deductions
V	________					
VI	________					
VII	1 2				No Yes	0 ½ 1
VIII	1 2	1 2			No Yes	0 ½ 1
IX	1 2	1 2			No Yes	0 ½ 1
X	1 2	1 2			No Yes	0 ½ 1
XI	1 2	1 2			No Yes	0 ½ 1
XII	1 2 3 4	1 2 3 4			No Yes	0 ½ 1 1½ 2
XIV	1 2 3 4	1 2 3 4			No Yes	0 ½ 1 1½ 2
Adult	1 2 3 4	1 2 3 4			No Yes	0 ½ 1 1½ 2

Max. Score: __________ **Deductions:** _____________ **TA:** ____________ **TQ:** _____________

References

Carlozzi, N. (2011). Porteus Maze. In *The Encyclopedia of Clinical Neuropsychology*. (pp. 1964-1966). New York: Springer.

David, K. H. (1974). Cross-cultural uses of the Porteus maze. *The Journal of Social Psychology, 92*(1), 11-18.

Gow, L., & Ward, J. (1982). The Porteus maze test in the measurement of reflection/impulsivity. *Perceptual and Motor Skills, 54*, 1043-1052.

Krikorian, R., & Bartok, J. A. (1998). Developmental data for the Porteus Maze Test. *Clinical Neuropsychologist, 12*(3), 305-310.
